# Changes in the Suitable Habitat of the Smoke Tree (*Cotinus coggygria* Scop.), a Species with an East Asian–Tethyan Disjunction

**DOI:** 10.3390/plants14040547

**Published:** 2025-02-10

**Authors:** Zichen Zhang, Xin Yan, Chang Guo, Wenpan Dong, Liangcheng Zhao, Dan Liu

**Affiliations:** 1 School of Ecology and Nature Conservation, Beijing Forestry University, Beijing 100083, China; zhangzichen@bjfu.edu.cn (Z.Z.); Yanxin999@bjfu.edu.cn (X.Y.); guochang@bjfu.edu.cn (C.G.); wpdong@bjfu.edu.cn (W.D.); 2 Museum of Beijing Forestry University, Beijing Forestry University, Beijing 100083, China; 3 Shandong Provincial Center of Forest and Grass Germplasm Resources, Ji’nan 250102, China

**Keywords:** *Cotinus coggygria*, species distribution models, environment variables, suitable habitat, glacial refugia

## Abstract

The smoke tree (*Cotinus coggygria* Scop.) is a woody species mainly distributed in the Mediterranean region and East Asia, known for its high ecological and ornamental value. Investigation of changes in suitable habitats under different conditions can provide valuable insights with implications for predicting the distribution of *C. coggygria*. In this study, we employed a MaxEnt model to simulate the current, historical, and future suitable habitat of *C. coggygria* using distribution records and environmental variables. The results indicated that climatic variables had a much stronger impact on the suitable habitat of this species compared with soil and topographic variables, and bio11 (mean temperature of the coldest quarter) and bio12 (annual precipitation) played particularly important roles in determining the suitable habitat. The core distribution of *C. coggygria* exhibited an East Asian–Tethyan disjunction. During the glacial period (Last Glacial Maximum), *C. coggygria* in Europe was concentrated in the glacial refugia in southern Europe; its range was substantially smaller during the glacial period than during interglacial periods (mid-Holocene). In contrast, *C. coggygria* in East Asia survived in regions similar to those of the interglacial period. Future climate change led to a gradual northward expansion of suitable habitats for *C. coggygria*, and the area of suitable habitat was substantially larger in Europe than in East Asia. There were significant differences among the four climate scenarios in Europe, with minimal variation in East Asia. Our findings provide valuable insights into the contrasting effects of climate change on European and East Asian populations of *C. coggygria*, which enhances our understanding of Eurasian species with discontinuous distributions.

## 1. Introduction

The East Asian–Tethyan disjunction refers to the discontinuous distribution of species between East Asia and the Mediterranean region, including Mediterranean Europe and adjacent Africa [1]. This distributional pattern, which is often examined in biogeographical and phylogenetic studies, has been documented in many plant and animal species [2,3,4,5]. Previous studies have suggested that the cause of the East Asian–Tethyan disjunction is related to climate change associated with geological changes, which includes the drought caused by the gradual retreat of the Tethys Sea during the Eocene [6] and the monsoon circulation associated with the violent uplift of the Qinghai–Tibetan Plateau [7]. Given that climate change has had a significant influence on the East Asian–Tethyan disjunction, studies of changes in distributions under the climatic background of different historical glacial periods and future climatic scenarios are needed to clarify the distributions of species.

Numerous studies have demonstrated that many variables affect species distributions [8]. For example, temperature and precipitation within the tolerance range of a species can affect its geographic range [9]. In addition, soil, which serves as a crucial medium for plant growth, provides water and nutrients to plants, which affects the ecological niche of species [10]. Soil properties can serve as indicators of habitat conditions and human activities at small scales and thus provide valuable information for simulating species distributions [11]. Soil represents an additional environmental variable that drives changes in the distributions of plant species. Topography plays a significant role in shaping species distributions [12], and elevation, aspect, and slope have been identified as key topographic variables affecting species diversity [13,14].

Species distribution models (SDMs), also known as ecological niche models, are widely used in biogeography [15], ecology, conservation biology, and wildlife management [16]. SDMs describe the relationship between species and the environment and can be used to predict spatial distributions via statistical methods, machine learning models, species location information or diversity data, and environmental variables that affect habitat suitability [17,18]. Several species distribution models have been used to simulate species niches, including MaxEnt (Maximum Entropy modeling) [19], Random Forest [20], and Boosting Regression Tree [21]. MaxEnt is the most widely used SDM due to its wide range of applications and high accuracy of simulations based on small sample sizes [22]. SDMs have been widely applied in many fields, such as biogeography, species diversity, and global climate change [15].

The smoke tree (*Cotinus coggygria* Scop.) is the most extensively cultivated species, and it also has the widest natural distribution in the genus *Cotinus* (Anacardiaceae) [23]. *C. coggygria* spread to the Mediterranean from South-Central Europe; it then crossed the continent, including the Himalayas, and entered China. During this dispersal process, it has adapted to different habitats and diversified in the phenotype [23,24]. Previous phylogenetic studies indicate that *Cotinus* and its sister genus *Pistacia* diverged in the middle and late Eocene (38.41 Ma), and it was not until the early Miocene (17.91 Ma) that *Cotinus* species began to diverge [25] and subsequently occupy Eurasia [23], which is consistent with the biogeography of *Pistacia* [2]. Because of its high ecological value and ornamental value, *C. coggygria* has been widely planted in Asia, Europe, and North America [23,26]. *C. coggygria* is most commonly cultivated using stem cuttings [24]. Previous studies have confirmed that the differentiation between *C. coggygria* populations is related to environmental differences [27,28], and precipitation is a key driver of genetic differentiation [28]. The potential distribution of this species in China under current conditions and during the Last Glacial Maximum (LGM) was simulated using MaxEnt [27]. However, these studies have only investigated the population differentiation of *C. coggygria* in the Yellow River Basin in Central and Northern China; the suitability of other regions on the Eurasian continent for *C. coggygria* thus remains unclear. The effects of soil and topographic variables should also be considered in analyses of the potential distribution of *C. coggygria*.

In this study, MaxEnt models were used to simulate the suitable habitat for *C. coggygria* under current conditions and during historical and future periods based on occurrence records and soil, topographic, and climatic variables. Specifically, we aimed to (1) identify the most important environmental variables affecting the distribution of *C. coggygria,* (2) characterize the current suitable habitat of *C. coggygria* in Eurasia, and (3) determine changes in the East Asian–Tethyan disjunction of *C. coggygria* between historical and future periods.

## 2. Materials and Methods

### 2.1. Collection and Processing of Distribution Records

A total of 1771 occurrence records of *C. coggygria* were obtained after selecting preserved specimen records with coordinates from the Global Biodiversity Information Facility (GBIF, https://www.gbif.org/, accessed on 24 October 2023) [29] and field-collected data obtained in nature. Duplicate records and records not located in Eurasia were excluded. The SDM Toolbox v 2.5 in ArcGIS v 10.8 was used to filter the data. SDMToolbox v 2.5 is a Python-based ArcGIS toolkit, which can simplify the complex processing required for species distribution modeling and geospatial analysis [30]. With SDMToolbox, only one distribution record was retained in each 10 km × 10 km grid to reduce errors caused by spatial autocorrelation. Finally, 335 effective occurrence records were obtained, of which GBIF data accounted for 94.3% and field-collected data accounted for 5.7% (Figure S1). The distribution range of field-collected data was largely consistent with the descriptions in the Flora of China. It covers the core distribution range in China and provides support for the East Asian portion of the GBIF data. All the occurrence records covered the distribution range of *C. coggygria* in previous studies [23,27,31].

### 2.2. Environmental Variable Selection

Data on 39 environmental variables (climatic variables, topographic variables, and soil variables) were obtained in this study (Table S1). The 19 current (1970–2000) climatic variables (bio1–bio19) were downloaded from WorldClim (https://worldclim.org/, accessed on 19 October 2023); the data had a resolution of 2.5 arc-min (5 km spatial resolution at the equator). Global elevation data, which had a resolution of 2.5 arc-min, were downloaded from WorldClim (https://worldclim.org/, accessed on 15 December 2023), and the slope and aspect were extracted using ArcGIS v 10.8. The 17 soil variables were extracted from the soil data in the World Soil Database (HWSD, https://www.fao.org/soils-portal/data-hub/en/, accessed on 16 December 2023). To make these data consistent with the climatic and topographic data, the resolution of the soil data was transformed from 30 arc-seconds to 2.5 arc-min using the Resample tool in ArcGIS v 10.8 in the WGS 1984 geographic coordinate system.

Due to the complex correlations among environmental variables, the 39 environmental variables were screened according to their correlations and contributions to reduce the influence of collinearity between variables on the prediction accuracy of the model [32]. First, the contribution rate of 39 environmental variables to the distribution of *C. coggygria* was determined by MaxEnt, and the contribution rates ranged from 0 to 1. Environmental variables with a contribution rate of less than 0.25 were removed. Second, correlation coefficients (r) of 39 environmental variables were calculated using ENMTools v 1.4.4 [33]. If |r| > 0.75 for a pair of environmental variables, the one with the higher contribution rate was selected. Finally, sixteen environmental variables, including six climatic variables, seven soil variables, and three topographic variables, were selected for model analysis based on the contribution rate and correlation coefficient (Figure S2). The contribution rate of climatic variables was the highest (59.2%), followed by topographic variables (9.6%) and soil variables (8.1%) (Figure S2a).

### 2.3. MaxEnt Simulations and Model Accuracy Evaluation

The current suitable habitat was modeled by MaxEnt 3.4.1 using 335 *C. coggygria* occurrence records and 16 environmental variables. A jackknife test was performed to measure the importance of each environmental variable. Model training was performed using 75% of the distribution data, and the remaining 25% of the data were used for model testing [34]. Model values were output in logistic format [35]. The max number of background points was set to 10,000. The maximum iterations were set to 5000, which means that the training will stop after 5000 iterations of the optimization algorithm. The running process was repeated 10 times with bootstrap as the replicated run type and an average was taken as the result. Other settings were set to their default values.

The accuracy of the model was evaluated using the area under the receiver operating characteristic (ROC) curve (AUC), which ranges from 0 to 1. We used the average test AUC value of 10 repeated runs to evaluate the model accuracy in this study. Higher AUC values indicate higher performance [36], and the simulation results were considered highly accurate when AUC > 0.9.

### 2.4. Classification of Suitable Habitat

Habitat suitability is expressed by a value between 0 and 1 in the MaxEnt result; values closer to 1 indicate higher suitability. We used the Reclassify tool in ArcGIS v 10.8 to classify the whole research region into four suitability grades. For its equality, objectivity, and discriminability [37], the mean maximum test sensitivity plus specificity logistic threshold of 10 replicates in each simulation result was used to distinguish between suitable and unsuitable habitats. Areas with values greater than the mean maximum test sensitivity plus specificity logistic threshold were designated as suitable habitat, and areas with values less than the threshold were designated as unsuitable habitat. Suitable habitats were divided into lowly suitable habitats, moderately suitable habitats, and highly suitable habitats (Table S2). The Zonal Geometry as Table tool in ArcGIS v 10.8 was used to calculate the area of suitable habitat under the Goode Homolosine projection. The spatial distribution and area of habitat with each suitability level were obtained for comparative analysis.

### 2.5. Simulation of the Historical Suitable Habitat

For the historical period, including the Last Glacial Maximum and mid-Holocene (MH), we downloaded climatic data of the CCSM4 model from WorldClim (https://worldclim.org/, accessed on 19 October 2023) at a resolution of 2.5 arc-min. To reduce the influence of collinearity between variables on the prediction accuracy, we extracted the climatic variables from the 16 environmental variables that were retained according to their contribution rates and correlation coefficients in Section 2.2.

Historical climatic variables were used as projection layers based on the occurrence records and current climatic variables. This historical model was built based on the same MaxEnt parameters and classification method as the simulation of the current suitable habitat.

### 2.6. Simulation and Migration of the Future Suitable Habitat

For future periods (2050s, 2070s, and 2090s), we downloaded climatic variables extracted in 2.2 from WorldClim (https://worldclim.org/, accessed on 19 October 2023) with a resolution of 2.5 arc-min. Considering the uncertainty in future climate projections, we calculated the arithmetic averages of three general circulation models (GCMs) (ACCESS-CM2, BCC-CSM2-MR, and GISS-E2-1-G) to take into account various future models [32]. Given that emission scenarios are affected by various socioeconomic assumptions, the predicted values of climatic variables varied under the different scenarios. In each GCM, four scenarios (SSP126, SSP245, SSP370, and SSP585) were examined, which combine representative concentration pathways (RCPs) and shared socioeconomic pathways (SSPs) to drive Coupled Model Intercomparison Project (CMIP6) climate models [38,39]. SSPs show future development trajectories related to human society’s ability to cope with climate change [39]. SSP1 represents a sustainable development society with a high degree of environmental friendliness, SSP2 represents a society with a more moderate development mode, SSP3 represents a society with slow economic growth and highly unbalanced development due to high emissions and rapid population growth, and SSP5 represents a society lacking climate strategies with high fuel consumption and few alternative energy sources [40]. RCPs refer to radiative forcing values, which range from 2.6 to 8.5 W/m^2^ by 2100 and reflect land use and atmospheric emissions [41]. As in the simulation of the historical suitable habitat, future climatic variables were used as projection layers in MaxEnt.

To clarify spatial changes in the suitable habitat under different future climate scenarios, the Mean Center tool in ArcGIS [42] was used to calculate the distribution center of the suitable habitat in each climate scenario; the center point was then converted into a line to show the migration direction and distance.

## 3. Results

### 3.1. Environmental Variable Selection

The AUC values of the ROC curves for the MaxEnt models based on test data were all above 0.97 after 10 repetitions. The average of these 10 values was 0.9832 (Figure S3a), which indicates that the model was effective in simulating the suitable habitat for *C. coggygria*.

The jackknife test results showed that climatic variables were more important than soil variables and topographic variables. The mean temperature of the coldest quarter (bio11) was the most significant variable in the model, as it showed the highest test gain when used in isolation; the omission of this variable also resulted in the largest reduction in test gain among all variables. The four variables with the highest test gain when used in isolation were all climatic variables, including the mean temperature of the coldest quarter (bio11), temperature seasonality (standard deviation ×100) (bio4), annual precipitation (bio12), and mean diurnal range (bio2). The first two variables were temperature factors (Figure S3b). According to the response curves, the optimal values of bio11, bio4, bio12, and bio2 were 3.43 °C, 671.67 °C, 688.42 mm, and 8 °C, respectively (Figure S3c–f).

### 3.2. Suitable Habitat Under Current Conditions

**Figure 1:**
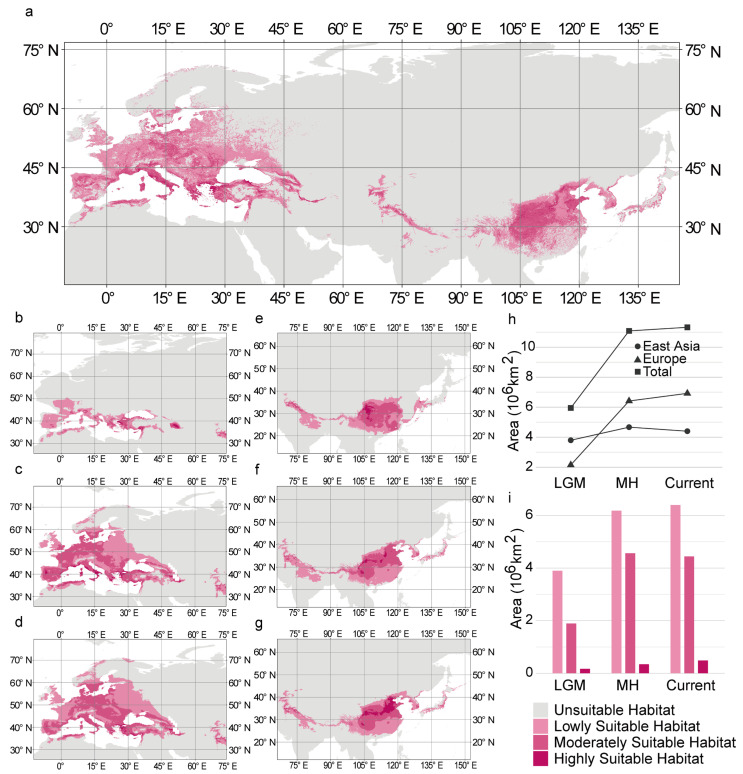
Historical changes in suitable habitat for *Cotinus coggygria.* (**a**) Current suitable habitat of *C. coggygria* modeled with 16 environmental variables. (**b**–**d**) The suitable habitat in Europe during the (**b**) Last Glacial Maximum, (**c**) mid-Holocene, and (**d**) current period. (**e**–**g**) The suitable habitat in East Asia during the (**e**) Last Glacial Maximum, (**f**) mid-Holocene, and (**g**) current period. (**h**) The area of suitable habitat during different periods. (**i**) The area of lowly suitable habitat, moderately suitable habitat, and highly suitable habitat during different periods.

The simulated results showed that the main suitable habitat for *C. coggygria* was widely distributed in East Asia and Europe, but a disjunction was present. Moderately suitable habitat and lowly suitable habitat were widely distributed from Southern to Central and even Northern Europe, as well as Southwest to North China, and highly suitable habitat was concentrated along the northern coast of the Mediterranean and North China (Figure 1a). The total area of suitable habitat was 8.47 × 10^6^ km^2^; the area of lowly suitable habitat, moderately suitable habitat, and highly suitable habitat accounted for 68.84%, 29.39%, and 1.77% of the total area of suitable habitat, respectively.

### 3.3. Changes in the Distribution of Historical Suitable Habitat

The suitable habitat of *C. coggygria* was mainly distributed in East Asia and Europe in all periods examined, and the suitable habitat at high latitudes increased from the Last Glacial Maximum to the mid-Holocene and current period (Figure 1b–g). During the Last Glacial Maximum, the suitable habitat was restricted to the Mediterranean coast around 40° N. By the mid-Holocene, the suitable habitat expanded to higher latitudes in Europe, reaching a maximum latitude of 70° N, which is similar to its current distribution (Figure 1b–d). The distribution of suitable habitats in East Asia did not change much, and this was in contrast to the patterns observed in Europe. During the Last Glacial Maximum, the distribution of suitable habitat was between 20° N and 40° N, and it spread north of 40° N in the mid-Holocene. The range of suitable habitat in the current period did not differ much from that in the mid-Holocene; only a slight shift to higher latitudes was observed (Figure 1e–g).

Changes in the distribution of habitat in each suitability grade varied in Europe and East Asia. (Figure 1b–g). From the Last Glacial Maximum to the current period, the highly suitable habitat in Europe was consistently clustered in the southern margin of the entire suitable habitat at approximately 40° N. Moderately suitable habitat and lowly suitable habitat in Europe expanded to higher latitudes (Figure 1b–d). In East Asia, most of the highly suitable habitats gradually expanded to North China. The moderately suitable habitat was clustered around the highly suitable habitat from Southwest China to North China. As the northward migration of moderately suitable habitat expanded, the southern part of the moderately suitable habitat transformed into a lowly suitable habitat (Figure 1e–g).

### 3.4. Changes in the Area of Historical Suitable Habitat

The total area of suitable habitat in the Last Glacial Maximum was 5.95 × 10^6^ km^2^, and this increased to 11.09 × 10^6^ km^2^ in the mid-Holocene, which was 1.86 times higher than that in the Last Glacial Maximum. An increase in the area of suitable habitat of 0.24 × 10^6^ km^2^ was observed from the mid-Holocene to the current period (Figure 1h, Table S2). The area of suitable habitat in Europe was much smaller than that in East Asia during the Last Glacial Maximum. By the mid-Holocene, the area of suitable habitat in Europe increased by 197.95% and exceeded that in East Asia. From the mid-Holocene to the current period, the area of suitable habitat in Europe increased, and that in East Asia began to decrease (Figure 1h, Table S2).

Changes in the area of habitat in each suitability grade varied in Europe and East Asia. In both Europe and East Asia, the proportion of lowly suitable habitat was the largest, followed by moderately suitable habitat and highly suitable habitat, in all periods (Figure 1i, Table S2). The proportion of highly suitable habitat during the Last Glacial Maximum and the current period was lower in Europe than in East Asia, and it was higher in Europe than in East Asia only during the mid-Holocene. Although the total area of suitable habitat in Europe in the current period was large, only the proportion of moderately suitable habitats and lowly suitable habitats was larger; the proportion of highly suitable habitats was much smaller in Europe than in East Asia (Table S2).

### 3.5. Suitable Habitat of C. coggygria Under Future Climate Change

**Figure 2:**
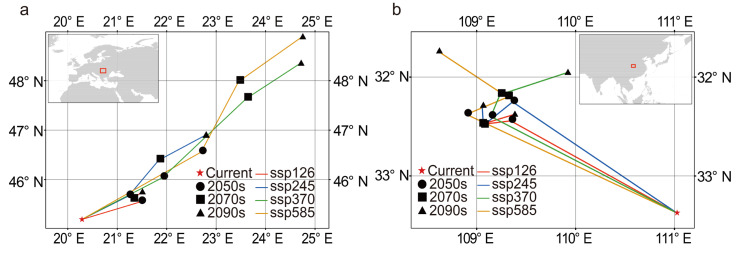
The migration of the center of suitable habitat in (**a**) Europe and (**b**) East Asia.

**Figure 3:**
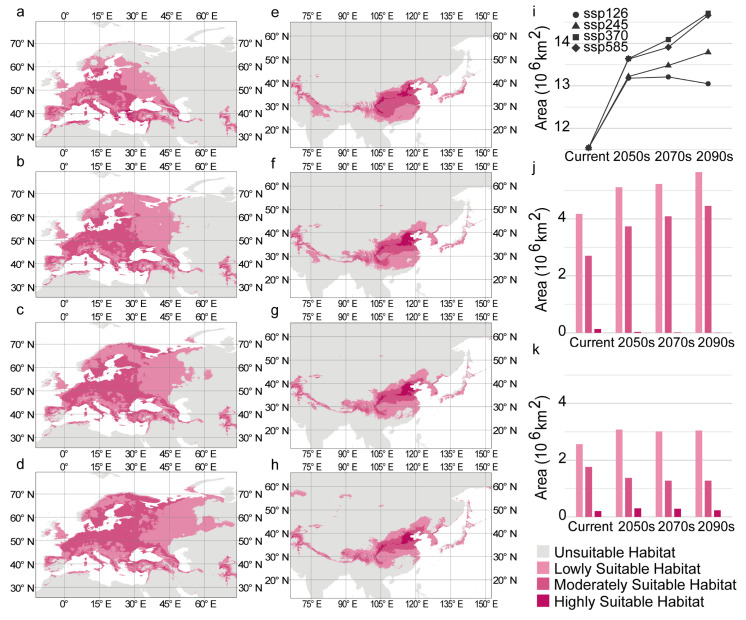
Suitable habitat of *C. coggygria* under the future climate. (**a**–**d**) The suitable habitat in Europe during the (**a**) current period, (**b**) 2050s, (**c**) 2070s, and (**d**) 2090s under SSP 585. (**e**–**h**) The suitable habitat in East Asia during the (**e**) current period, (**f**) 2050s, (**g**) 2070s, and (**h**) 2090s under SSP585. (**i**) Changes in the area of suitable habitat in different periods under SSP126, SSP245, SSP370, and SSP585. (**j**,**k**) The area of lowly suitable habitat, moderately suitable habitat, and highly suitable habitat in (**j**) Europe and (**k**) East Asia during different periods under SSP585.

The distribution of suitable habitats for *C. coggygria* changed significantly over time. The distribution center of the suitable habitat in Europe and East Asia moved northward from the current period to the 2090s (Figure 2a–b). Changes in the distribution of suitable habitat were highly significant under SSP585, which is the scenario with the highest emissions. The suitable habitat in Europe rapidly expanded into most of Europe, and the southern boundary of the suitable habitat remained almost unchanged (Figure 3a–d). The main suitable habitat in East Asia gradually shifted to Northern China, and this caused wide areas of suitable habitat in Southern China to be transformed into unsuitable habitat (Figure 3e–h); this contrasted with the patterns of change observed over time in Europe. Regardless of the specific scenario, the highly suitable habitat in both Europe and East Asia was mainly distributed from 30° N to 40° N. The moderately suitable habitat and lowly suitable habitat below 30° N gradually decreased (Figure 3a–h). The trends observed in the other scenarios were similar to those observed under SSP585.

The total area of suitable habitat under most scenarios gradually increased over time and was much larger than the area of suitable habitat under current conditions (Figure 3i). The average total area reached 14.05 × 10^6^ km^2^ in the future, which was 1.22 times higher than that under current conditions (Table S3). Under SSP585, the total area of suitable habitat in Europe, more than 99% of which comprised moderately suitable habitat and lowly suitable habitat, was always larger than that in East Asia (Table S3). The total area of suitable habitat in Europe substantially increased from the current period to the 2090s (increasing from 7.01 × 10^6^ km^2^ to 10.11 × 10^6^ km^2^), and the area of highly suitable habitat decreased to 0.00724 × 10^6^ km^2^, which only accounted for 0.07% of the area of suitable habitat in Europe (Figure 3j, Table S3). In East Asia, the total area of suitable habitat fluctuated steadily between 4.52 × 10^6^ km^2^ and 4.75 × 10^6^ km^2^ (Table S3). The area of highly suitable habitat was larger in East Asia than in Europe and was more than 0.2 × 10^6^ km^2^ in all periods, which accounted for 4.56–6.37% of the area of suitable habitat in East Asia (Figure 3k, Table S3). The trends under other scenarios were similar to those observed under SSP585 (Table S3).

### 3.6. Variation in Suitable Habitat Under Different Future Climate Scenarios

**Figure 4:**
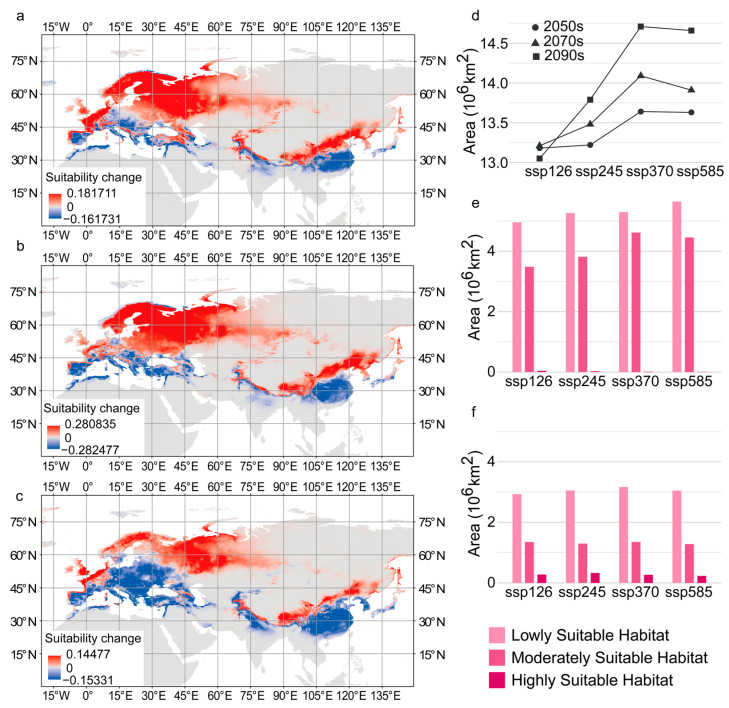
Suitable habitat of *C. coggygria* under different SSPs in the future. (**a**–**c**) Suitability changes (**a**) from SSP126 to SSP245, (**b**) from SSP245 to SSP370, and (**c**) from SSP370 to SSP585 in the 2090s. (**d**) Changes in the area of suitable habitat under different SSPs in the 2050s, 2070s, and 2090s. (**e**,**f**) Areas of lowly suitable habitat, moderately suitable habitat, and highly suitable habitat in (**e**) Europe and (**f**) East Asia under different SSPs.

The suitability of habitat for *C. coggygria* in the same areas varied among scenarios. In the 2090s, the habitat suitability values for *C. coggygria* increased in the northern part of the entire range of suitable habitats and decreased in the southern part of the entire range of suitable habitats as expected emissions increased; this indicates that the suitable habitat increased in the north (Figure 4a–c). The trends in other periods were similar to those observed in the 2090s.

The suitable habitat area was larger under each future climate scenario than under current conditions (Figure 4d). The area of suitable habitat and the proportion of different habitat suitability grades under different climate scenarios varied greatly in Europe and varied little in East Asia (Table S3). In the 2090s, changes in the distribution of habitat in each suitability grade under different scenarios ranged from 0.18 × 10^6^ km^2^ and 1.62 × 10^6^ km^2^ in Europe, but all were less than 0.24 × 10^6^ km^2^ in East Asia (Figure 4e–f, Table S3). The trends in other periods were similar to those observed in the 2090s (Table S3).

## 4. Discussion

### 4.1. Differences in the Effects of Environmental Variables on C. coggygria

Six of the sixteen climatic variables screened contributed nearly 60% to the model (Figure S2b). This result was similar to the results of the jackknife test, in which the four environmental variables with the greatest effect on the MaxEnt model were all climatic variables (Figure S3b). This indicates that climate conditions played the most important role in determining the distribution of *C. coggygria*. Many previous SDM studies have shown that the contribution of climatic variables was greater than that of soil variables and topographic variables [43,44,45].

Previous studies have shown that *C. coggygria* is a locally adapted species with strong responses to precipitation and temperature conditions [28,46]. In this study, our results suggested that bio11 (mean temperature of coldest quarter) is a key variable determining the suitability of *C. coggygria*. Bio12 (annual precipitation) is also an important variable affecting the suitability of *C. coggygria* (Figure S3b). In previous studies of adaptive genetic variation in *C. coggygria*, bio12 was found to be significantly correlated with two alleles (Cc 025D and Cc 025F) in *C. coggygria*, and Cc 025F was also significantly correlated with precipitation variables (bio5, bio6, and bio8) [28]. In the models established based on historical, current, and future climate data, bio11 and bio12 were the variables with the highest contribution rate, which further verified the important effects of these two variables on the distribution of *C. coggygria*. Therefore, bio11 and bio12 have key effects on the suitability of habitat for *C. coggygria*.

Environmental stress induces responses at the cellular and molecular level in plants [47]. Low-temperature stress can make the extracellular water and water in the tracheary element freeze, resulting in cell dehydration, blocking water transport, and thus affecting the physiological metabolic processes of plant cells [48,49]. The important effect of the mean temperature of the coldest quarter on the suitable habitat reflects the sensitivity of *C. coggygria* to low-temperature stress, which can also explain the northward shift in the suitable habitat of *C. coggygria* under climate warming caused by increasing emissions in the future. Similar to low temperatures, insufficient precipitation can also limit physiological processes such as plant growth, development, and reproduction [47]. In this study, the relationship between the suitable habitat of *C. coggygria* and annual precipitation indicates that precipitation has a major effect on the suitability of *C. coggygria*.

### 4.2. Changes in the Distribution of C. coggygria

The distribution of *C. coggygria* has been discontinuous between Europe and East Asia since the Last Glacial Maximum (2.1 Ma) (Figure 1). The distribution of *C. coggygria* was affected by the glacial period, and changes in its distribution in East Asia and Europe were not consistent. Specifically, East Asia provides a habitat for many Tertiary relict species since it was less affected by ice sheets during the glacial period [50]. The mountains of Southwest China and Northern Vietnam in particular are considered long-term stable refugia because of their mild climatic conditions [51]. In this study, the survival range of *C. coggygria* in East Asia during the Last Glacial Maximum is not much different from that in the interglacial (mid-Holocene) and current period (Figure 1e–g), which was comparable to the simulation results obtained using the distribution data in China [27]. These survival ranges were consistent with observed patterns in China’s warm-temperate zone. In contrast to patterns observed in East Asia, during the Last Glacial Maximum, the area of suitable habitat in Europe was much lower than that during the interglacial period (mid-Holocene) and concentrated in Southern Europe (Figure 1b); it overlapped with the refugia of numerous temperate tree species during the Quaternary glacial period [52,53]. This result was consistent with the southern refugia hypothesis [54].

The suitable habitat of *C. coggygria* shifted to the north from the current period to the 2090s (Figure 2). This finding is consistent with the prediction of the future suitable habitat of many species located in Europe [55,56] and East Asia [57,58,59]. For example, the distribution of European Hop Hornbeam (*Ostrya virginiana*) in Europe will move northward over the next 60 years [55]. The potential habitat of *Ziziphus jujuba* in China will shift northeastward to adapt to global warming [59]. Future climate conditions are more suitable for the survival of *C. coggygria* compared with current conditions, and the suitable habitat is mainly located to the north of the current distribution. Specifically, the suitable habitat in Europe is rapidly expanding into most of Europe, and the main suitable habitat in East Asia is gradually moving to southwestern and northeastern China. Our results indicate that a northerly climate would be suitable for *C. coggygria* in the future. The results under different scenarios indicated that *C. coggygria* tends to occupy a wider area of suitable habitat and higher latitudes under higher emission climate scenarios (Figure 3). This is in contrast to the results of previous analyses of many species, such as *Cunninghamia lanceolata* [60] and *Sapindus mukorossi* [61], which indicate that the area of the suitable habitat was positively related to the environmental friendliness of the climate scenario. Our results revealed that future climate conditions will be more suitable for *C. coggygria* than current climate conditions.

## 5. Conclusions

The results of this study revealed the adaptation of *C. coggyria* to environmental variables and differences in the suitable habitat under different climatic conditions between Europe and East Asia. Climatic variables had a significantly stronger effect on the suitable habitat of this species than soil variables and topographic variables. Bio11 and bio12 were particularly important variables. *C. coggygria* exhibits a disjunct East Asian–Tethyan distribution, and its core distribution region was concentrated in the Mediterranean and East Asia. During the Last Glacial Maximum, *C. coggygria* in Europe was much lower than that during the interglacial period and concentrated in glacial refugia in Southern Europe, whereas *C. coggygria* in East Asia was present in regions similar to interglacial habitats. Under future climate scenarios, the suitable habitat of *C. coggygria* gradually expand northward. As the climate scenarios became more extreme, the suitable habitat of *C. coggygria* shifted northward. The overall area of suitable habitat was larger in Europe than in East Asia. The area of highly suitable habitat was smaller in Europe than in East Asia, and the area of suitable habitat in Europe significantly differed under the four scenarios.

## Data Availability

The original contributions presented in the study are included in the article/Supplementary Materials, further inquiries can be directed to the corresponding author.

## Supplementary Materials

The following supporting information can be downloaded at: https://www.mdpi.com/article/10.3390/plants14040547/s1. Figure S1: The 335 distribution records of *Cotinus coggygria*; Figure S2: Environmental variable screened. (a) Correlation and (b) contribution rate of 16 environmental variables involved in simulating; Figure S3: MaxEnt Simulations and Model Accuracy Evaluation. a Receiver operating characteristic (ROC) curves. b jackknife test of variable importance. c-r MaxEnt model response curves of 16 environmental variables; Table S1: The environmental variables used in this study; Table S2: The classification range of suitable habitat in each model; Table S3: Area of historical suitable habitat of each grade (10^6^ km^2^); Table S4: Area of future suitable habitat of each grade (10^6^ km^2^).
