# Supplementary material for: Changes in the Suitable Habitat of the Smoke Tree (Cotinus coggygria Scop.), a Species with an East Asian–Tethyan Disjunction
Source: Plants (Basel). 2025 Feb 10;14(4):547. doi: 10.3390/plants14040547 (PMC11859633; doi:10.3390/plants14040547)
Supplement: Supplementary file 1 [file plants-14-00547-s001.zip › plants-3470026-supplementary.pdf]

Supplementary Materials for

Changes in the suitable habitat of the smoke tree (*Cotinus coggygria*), a species with an East Asian-Tethyan disjunction

Zichen Zhang[1], Xin Yan[1], Chang Guo[1], Wenpan Dong[1], Liangcheng Zhao[2*], Dan Liu[3*]

[1] School of Ecology and Nature Conservation, Beijing Forestry University, Beijing 100083, China

[2] Museum of Beijing Forestry University, Beijing Forestry University, Beijing 100083, China

[3] Shandong Provincial Center of Forest and Grass Germplasm Resources, Ji'nan 250102, China

* Corresponding authors: Liangcheng Zhao: lczhao@bjfu.edu.cn

Dan Liu: 1821618@163.com

Supplementary figures

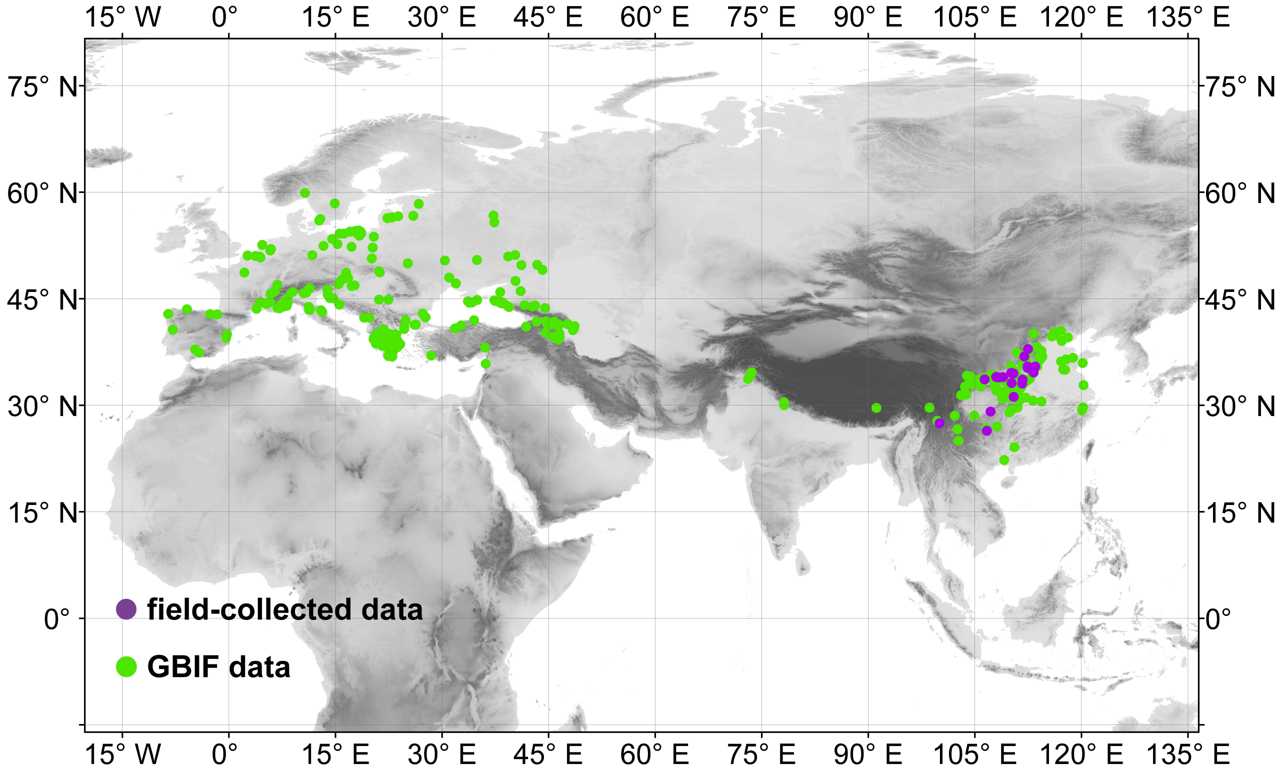

Figure S1 The 335 distribution records of *Cotinus coggygria.*

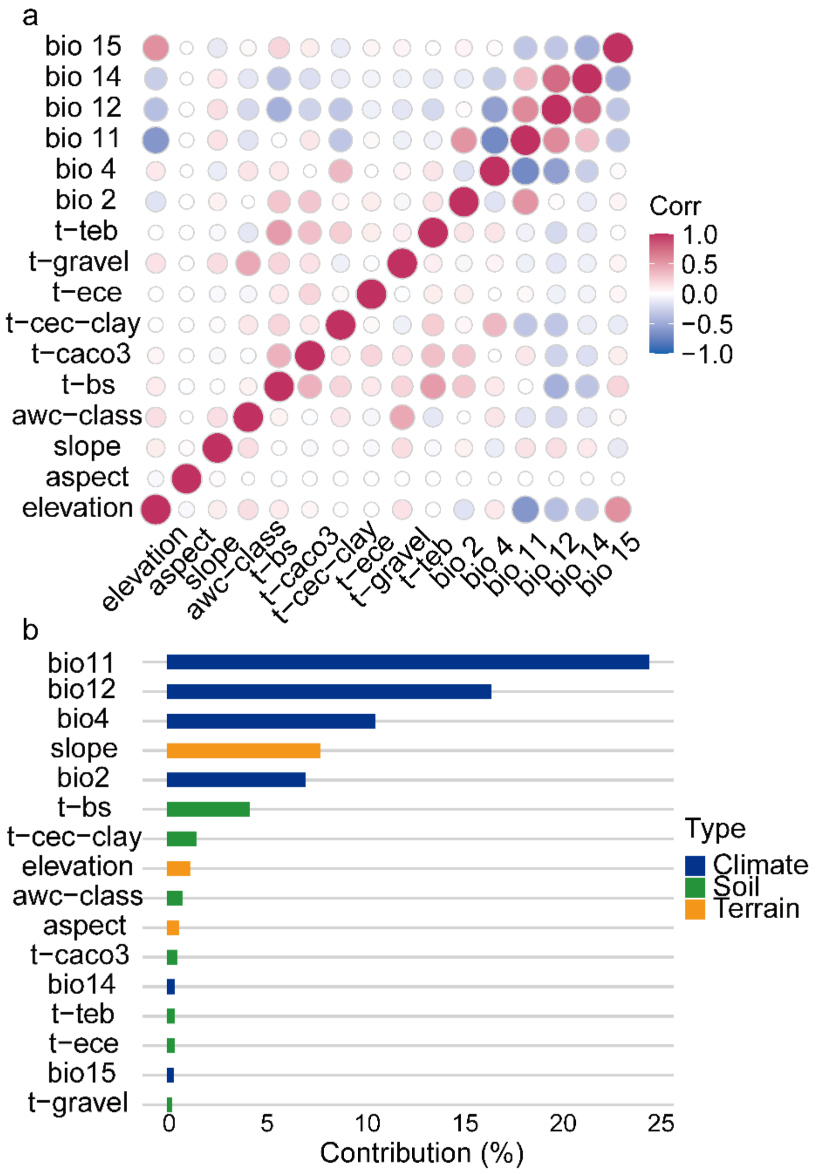

Figure S2 Environmental variable screened. (a) Correlation and (b) contribution rate of 16 environmental variables involved in simulating.

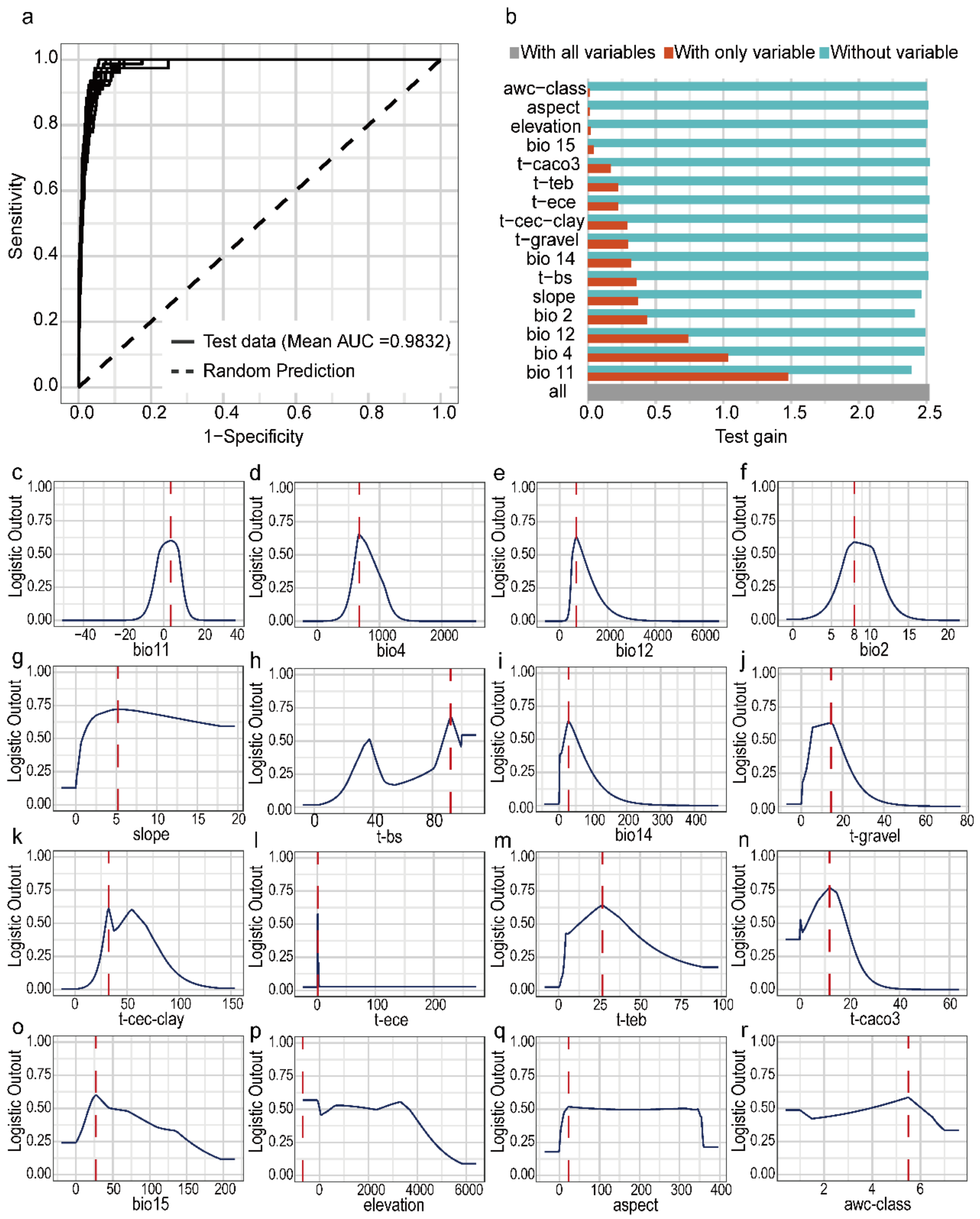

Figure S3 MaxEnt Simulations and Model Accuracy Evaluation. **a** Receiver operating characteristic (ROC) curves. **b** jackknife test of variable importance. **c-r** MaxEnt model response curves of 16 environmental variables.

Supplementary tables

Table S1 The environmental variables used in this study.

Variable	Discribution	Type	Variable	Discribution	Type
**bio1**	Annual Mean Temperature		**t-sand**	Topsoil Sand Fraction	
**bio2**	Mean Diurnal Range		**t-silt**	Topsoil Silt Fraction	
**bio3**	Isothermally (BIO2/BIO7) (* 100)		**t-clay**	Topsoil Clay Fraction	
**bio4**	Temperature Seasonality (standard deviation *100)		**t-usda-tex-class**	Topsoil USDA Texture Classification	
**bio5**	Maximum Temperature of Warmest Month		**t- bulk-density**	Topsoil Bulk Density	
**bio6**	Minimum Temperature of Coldest Month		**t-oc**	Topsoil Organic Carbon	
**bio7**	Temperature Annual Range (Bio5-Bio6)		**t-ph-h_2o**	Topsoil pH (H_2O)	
**bio8**	Mean Temperature of Wettest Quarter		**t-cec-clay**	Topsoil CEC (clay)	Soil
**bio9**	Mean Temperature of Driest Quarter	Climate	**t-cec-soil**	Topsoil CEC (soil)	
**bio10**	Mean Temperature of Warmest Quarter		**t-bs**	Topsoil Base Saturation	
**bio11**	Mean Temperature of Coldest Quarter		**t-teb**	Topsoil TEB	
**bio12**	Annual Precipitation		**t-$caco_3$**	Topsoil Calcium Carbonate	
**bio13**	Precipitation of Wettest Period		**t-$caso_4$**	Topsoil Gypsum	
**bio14**	Precipitation of Driest Period		**t-esp**	Topsoil Sodicity (ESP)	
**bio15**	Precipitation Seasonality (coefficient of variation)		**t-ece**	Topsoil Salinity (Elco)	
**bio16**	Precipitation of Wettest Quarter		**awc-class**	Available Water Content Range	
**bio17**	Precipitation of Driest Quarter		**elevation**	Elevation	
**bio18**	Precipitation of Warmest Quarter		**aspect**	Aspect	Topography
**bio19**	Precipitation of Coldest Quarter		**slope**	Slope	
**t-gravel**	Topsoil Gravel Content	Soil			

Table S2 The classification range of suitable habitat in each model.

	Unsuitable habitat	Lowly suitable habitat	Moderately suitable habitat	Highly suitable habitat
**Current model**	0–0.1111	0.1111–0.4074	0.4074–0.7037	0.7037–1
**Historical model**	0–0.0941	0.0941–0.3961	0.3961–0.6980	0.6980–1
**Future model**	0–0.1087	0.1087–0.4058	0.4058–0.7029	0.7029–1

Table S3 Area of historical suitable habitat of each grade (10^6 km^2).

	**Lowly Suitable Region**		**Moderately Suitable Region**		**Highly Suitable Region**	
	Europe	East Asia	Europe	East Asia	Europe	East Asia
**LGM**	1.795	2.102	0.338	1.549	0.020	0.149
**MH**	3.256	2.926	2.930	1.632	0.230	0.114
**Current**	3.797	2.603	3.030	1.411	0.098	0.389

Table S4 Area of future suitable habitat of each grade (10^6 km^2).

		Lowly Suitable Region		Moderately Suitable Region		Highly Suitable Region	
		Europe	East Asia	Europe	East Asia	Europe	East Asia
**Current**		4.176	2.561	2.703	1.761	0.131	0.207
**2050s**	**ssp126**	5.051	2.944	3.458	1.401	0.039	0.285
	**ssp245**	4.924	2.977	3.567	1.382	0.038	0.328
	**ssp370**	5.200	3.132	3.577	1.419	0.037	0.278
	**ssp585**	5.113	3.073	3.739	1.373	0.031	0.302
**2070s**	**ssp126**	5.158	2.923	3.457	1.352	0.037	0.283
	**ssp245**	5.104	2.999	3.679	1.359	0.032	0.310
	**ssp370**	5.316	3.136	3.954	1.349	0.022	0.317
	**ssp585**	5.227	3.013	4.091	1.278	0.014	0.288
**2090s**	**ssp126**	4.961	2.930	3.487	1.349	0.041	0.281
	**ssp245**	5.267	3.051	3.819	1.295	0.026	0.330
	**ssp370**	5.296	3.167	4.619	1.348	0.013	0.268
	**ssp585**	5.644	3.043	4.458	1.277	0.007	0.233
